# Impact of Hospital Visitor Restrictions on Racial Disparities in Obstetrics

**DOI:** 10.1089/heq.2020.0073

**Published:** 2020-12-03

**Authors:** Alexandra Norton, Tenisha Wilson, Gail Geller, Marielle S. Gross

**Affiliations:** ^1^ Johns Hopkins University School of Medicine, Baltimore, Maryland, USA.; ^2^ Department of Gynecology and Obstetrics, Johns Hopkins University School of Medicine, Baltimore, Maryland, USA.; ^3^ Berman Institute of Bioethics, Johns Hopkins University School of Medicine, Baltimore, Maryland, USA.

**Keywords:** obstetrics, health disparities, COVID-19

## Abstract

Racial disparities in both obstetrics and COVID-19 are well documented. Troublingly, implicit biases and related testimonial injustice potentiate adverse outcomes for women of color whose voices and concerns have been historically discredited by the medical establishment. In the context of COVID-19, the restriction of hospital visitors for infection prevention and control in a labor and delivery setting may disproportionately burden black women by eliminating or severely limiting access to essential in-person advocacy, which threatens to exacerbate existing disparities in maternal and neonatal outcomes. The potential disproportionate impact of visitor restrictions on women of color should inform the ongoing pandemic response.

## Introduction

Racial and ethnic disparities in obstetrical outcomes in the United States have been well documented. Non-Hispanic black women are three to four times more likely to die from a pregnancy-related cause than non-Hispanic white women.^1^ Although these disparities are not fully understood, key factors include access to prenatal care, underlying comorbidities, socioeconomic status, and chronic stress exposure due to discrimination and structural racism.^2,3^ These disparities may represent inequities, insofar as they are influenced by implicit biases within the health care system. This may further result in testimonial injustice, where a patient's statement or description is not treated as credible due to underlying prejudice.^4^ For example, reported symptoms may be dismissed or discounted, impeding needed care to a potentially deadly effect.^3,5^

Disease outbreaks tend to expose and increase underlying health disparities. Significant disparities are already seen in COVID-19, as the black population has a disproportionate share of infections, hospitalizations, and deaths in a majority of states.^6^ Data available for pregnant women have followed suit, with minority women disproportionately becoming infected with SARS-CoV-2, the virus that causes COVID-19.^7^

The long-established maternal health disparities and quickly emerging disparities in COVID-19 intersect in the U.S. obstetric in-patient setting. We discuss how infection prevention and control (IPC) required restriction of hospital visitors during labor and delivery may be especially harmful to minority and underserved women. Visitor restriction compromises access to critical health advocates, thereby threatening to reinforce and exacerbate the health effects of underlying structural racism. This article focuses on non-Hispanic black women, but the trends identified are likely to occur more broadly among other underserved patient populations.

## Hospital Visitor Restrictions in Obstetric Settings During COVID-19

Hospitals concentrate infectious risk, and stewardship of scarce resources, including personal protective equipment (PPE) and health care workers, is paramount. Furthermore, asymptomatic transmission and insufficient testing pose significant challenges for limiting the spread of SARS-CoV-2. In response, hospitals around the United States largely eliminated visitors in in-patient settings. Current recommendations advise health care facilities that only visitors essential for patients' “physical or emotional well-being and care” be permitted, and visitors have been deemed nonessential in most in-patient settings.^8^ The primary exceptions have been for labor and delivery, pediatric intensive care, and end-of-life care. “Minimum necessary” visitors are recommended in labor and delivery settings, generally translating to one visitor.^9^

Visitors provide critical patient support and advocacy. Engagement of families has been shown to improve patient outcomes and care quality.^10^ A Cochrane meta-analysis found that women receiving continuous in-person support during labor are more likely to have a spontaneous vaginal delivery and avoid interventions including operative vaginal or cesarean delivery.^11^ Visitors' advocacy becomes particularly important for those facing increased risk of testimonial injustice by literally strengthening these patients' voices, whose reported symptoms may fall on deaf ears.^3,4^ Outside of COVID-19, recognition of the distinct social and health benefits of visitors for labor and delivery has led hospitals to eliminate traditional restrictions on visitor numbers or hours.

Consistent with national recommendations during the pandemic, many U.S. hospitals have implemented a policy limiting women admitted for birth to one visitor, with several accompanying restrictions. This well-intentioned policy has introduced considerable strain on women and their loved ones, with potential disproportionate burdens for black women. For example, black women are more likely than white women to have previously received doula support and to desire it for future pregnancies.^12^ Visitor restriction policies typically classify doulas as visitors, regardless of professional designation, forcing women to choose between the evidence-based benefits of doula support and their partner or other personal support persons.^13,14^

Some hospitals do not permit any visitors if the parturient tests positive for SARS-CoV-2, with the Society for Maternal-Fetal Medicine recommending that visitors be “restricted or eliminated” for women who test positive.^15,16^ Given the prevalence of asymptomatic infections, increased testing capacity, and presumed vulnerability of pregnant women, many hospitals have moved to universally testing women admitted for delivery. Black women have been identified as more likely to serve as essential workers, increasing their exposure to the virus and likelihood of potential deprivation of critical intrapartum support.^17^ Indeed, the Centers for Disease Control and Prevention Morbidity and Mortality Weekly Report (MMWR - CDC) reports that nearly one quarter of pregnant women infected with SARS-CoV-2 are black, >50% greater than their frequency in the general population.^7^ This means black women have been more likely to labor, deliver, and recover alone during the pandemic.

Additional restrictions for women who test negative may include permitting only single entry for the visitor and prohibiting swapping one visitor for another.^14,15^ Continuous support thus requires visitors to have the financial and logistical flexibility to remain in the hospital for several days. This may be more difficult for underserved minorities who disproportionately work in jobs without paid leave or scheduling adjustments, or without the ability to work remotely.^18,19^ Patients with other children must be able to secure childcare independent of their selected visitor, an undertaking made more challenging by social distancing measures and travel restrictions.

Having to select one visitor may prevent black women from benefiting from the unique advocacy that different support persons, such as those with a medical background or experience with childbirth, would typically provide. Decreased advocacy has varying ramifications over the course of the hospitalization. In the postpartum period, unaddressed warning signs directly increase risk for maternal morbidity and mortality.^20^ Support for breastfeeding and other needs, including caring for the newborn, may also be reduced and may subsequently increase the likelihood of the newborn being separated or receiving formula supplementation.

Visitor restriction policies have led to some women laboring alone for part or all of their hospitalization, with potential adverse medical and psychological effects. Testimonial injustice can lead to symptoms being disregarded and subsequent adverse clinical outcomes, a concern that is enhanced without the presence of a personal advocate.^3^ Furthermore, lack of support during delivery is a risk factor for birth trauma and postpartum post-traumatic stress disorder, which can have long-term psychological sequelae.^21^ Though a topic unto itself, the potential for maternal–infant separation for infection control purposes both compounds psychological stress and threatens short- and long-term health effects for women and infants if breastfeeding is undermined.^22^

Many women have expressed increased interest in out-of-hospital births due to COVID-related concerns including the possibility of SARS-CoV-2 infection, visitor restrictions, and infant separation.^23^ These concerns, against a background of appropriate fear and distrust of the health care system and greater COVID-related risks, may make black women especially likely to either delay presenting to the hospital with obstetric symptoms or plan on out-of-hospital birth during the pandemic.^24^ Though out-of-hospital birth may be a safe and empowering option, become even more appealing during COVID-19, underlying disparities in access to safe alternatives to hospital birth (i.e., geographically and financially accessible and affordable home birth attendants and birth centers) suggest greater potential for harms related to unattended delivery either en route or at home.^25^

## Conclusion

Eliminating or severely limiting visitors for parturients increases their vulnerability to health complications and imposes significant psychological stress. Black women are more likely to have COVID-19, increasing their likelihood of being denied any visitors during their birth hospitalization. Those permitted a single visitor may be disproportionately impacted by policies limiting a diversity of in-person support. In both cases, black women are more likely to be deprived of continuous, optimal support and advocacy, and they are more likely to experience an adverse physical or psychological outcome as a result. The potential for hospital IPC policy to unintentionally increase disparities in obstetric outcomes for black women is emblematic of the pervasive and compounding effects of implicit and explicit biases in the U.S. health care system.

## Recommendations and Future Directions

We recommend that hospitals consider taking the following actions to mitigate the harmful effects of COVID-related visitor restrictions on black parturients:

1. Doulas should be classified as health professionals, so that their presence is permitted without excluding other critical support persons.
2. Hospitals should implement accommodations when, per existing restrictions, a woman would labor without a support person of her choice. These necessitate appropriate safety precautions such as increased use of testing for visitors and adequate PPE provision, but could include permitting a visitor for SARS-CoV-2–positive women, allowing for swapping of visitors during hospitalizations that exceed a certain length, and considering multiple entry for visitors.
3. Hospitals should determine possible modifications for the frequency of in-person patient assessments for women who labor alone, to compensate for missing in-person support by increasing access to clinicians. To further compensate for missing support, in academic settings health professional students could serve as in-person patient advocates.

In this article we focus on implications of visitor restrictions for in-patient labor and delivery care. Further exploration of how visitor restrictions affect other women's health settings (e.g., prenatal care and gynecological oncology) is warranted. Other COVID-related policies, such as maternal–infant separation, must also be investigated to determine the extent to which COVID-19—and our response to it—may worsen health outcomes for black women and infants. Finally, we have focused on black women, but we must also consider these policies' impact on other minority populations. Careful consideration, surveillance, and ongoing evaluation of the implications of IPC policy in women's health settings are needed to help guard against the pandemic's risk of reinforcing racial disparities.

## Abbreviations Used

IPC: infection prevention and control
PPE: personal protective equipment
